# Environmental Penalties, Investor Attention and Stock Market Reaction: Moderating Roles of Air Pollution and Industry Saliency

**DOI:** 10.3390/ijerph19052660

**Published:** 2022-02-24

**Authors:** Hua Wu, Taiwen Feng, Wenbo Jiang, Ting Kong

**Affiliations:** 1 Inner Mongolia University of Finance and Economics, Hohhot 010070, China; breezewh@163.com; 2 School of Economics and Management, Harbin Institute of Technology (Weihai), Weihai 264209, China; 3 School of Economics and Management, Dalian University of Technology, Dalian 116081, China; jiangwenbo@mail.nwpu.edu.cn; 4 Business School, University of Shanghai for Science & Technology, Shanghai 201210, China; tingkong33@163.com

**Keywords:** environmental penalties, investor attention, stock market attention, air pollution, industry saliency

## Abstract

Despite the importance of environmental penalties in environmental enforcement, how and under what situations they impact stock market reaction is still unclear. Drawing on the theories of expectancy violation and attention driven, a conceptual model is built to explore how environmental penalty influences stock market reaction through investor attention. Furthermore, it is explored that the air pollution and industry saliency facilitate the indirect relationship between environmental penalty and investor attention. We empirically test this theoretical framework using a sample of 88 listed companies that received the environmental penalty. Up to 31 December 2020, a total of 88 A-share listed companies in Shanghai and Shenzhen stock exchanges were obtained as samples by collecting the announcement of environmental penalties of listed companies on Juchao Network. Furthermore Baidu index is taken as a proxy for investor attention in this study. Our findings reveal that investor attention plays mediating role in the relationship between environmental penalty and abnormal returns, while the direct effect of environmental penalty on stock market reaction has not been verified, thus, investor attention plays a complete mediating role between them. In addition, air pollution moderates the relationship between Environmental penalties and investor attention. The study found that enterprises in heavy pollution industries might suffer safety-in-numbers effect, which would weaken the directly negative impact of environmental penalties, and verified the moderating effect of industry saliency. These findings provide theoretical and practical implications for understanding how environmental penalties influence on stock market reaction.

## 1. Introduction

Environmental penalty has generated invaluable insights into how government to effectively control the environmental pollution of enterprises. Environmental penalty is critical tools for the government to effectively control environmental pollution because of their enforceability and operability [1]. Clearly, enterprises are more likely to invest in green initiatives and avoid punishment if they believe that these penalties make their shareholder wealth suffer huge losses. Alternatively, the lack of a significant negative impact of environmental penalty on stock market reaction is likely to suppress firm efforts and hold back environmental strategy toward a sustainable future [2,3,4].

The increasing importance of environmental penalty among pollution control practices is receiving considerable attention in academic researches [5]. A growing literature studies the reasons why government engage in environmental penalty and how it relates to shareholder wealth. In particular, the studies examined the relationship between environmental penalty and stock market reaction [6,7]. While these researches point toward a negative relationship between environmental penalty and abnormal return, little is known about whether and how this relationship has evolved over time.

However, despite the voluminous number of studies that have sought to address this issue, the cumulative evidence to date is largely inconclusive. As suggested by Capelle-Blancard and Petit [8], there is a direct negative impact of environmental penalties on market value. By contrast, others highlighted that environmental penalty have weak impacts on the stock market [9]. However, other researchers suggested that environmental penalties might have an immediate effect, which would slightly influence the stock market reaction in the short term, but cannot significantly change the environmental strategy of enterprises in the long term [2,10]. Although there is consistent evidence demonstrating that environmental penalty is related to stock market reaction, inconsistency of the effectiveness of the mechanism still exists, which provides an opportunity for us to further explore.

Moreover, the inconsistency of this mechanism was discussed based on the theory of expectancy violation and attention driven. In this study, the theories of derive hypotheses on how the relationship between environmental penalty and stock market reaction were extended. Then it is systematically investigated whether investor attention playing a key role to explain how the stock market reacted to these environmental penalties, and the difference in investor attention response to environmental penalties between heavy pollution industry and non-heavy pollution industry.

In this study, a total of 88 A-share listed companies in Shanghai and Shenzhen stock exchanges were obtained as samples by collecting the announcement of environmental penalties of listed companies on Juchao Network. Event Day 0 denoted the date of the announcement of environmental penalties. And the stock market effect within two trading weeks following the day of the announcement of punishment is analyzed in this study. The results indicate that stock market reaction to the environmental penalty is negative and significant on the first day after the announcement of punishment, but the negative impact gradually dissipated on the following day. Then it is analyzed the immediate and direct stock market reaction, which is clearly defined as the abnormal return of the first day after Day 0. The conceptual framework is built on the argument that investor attention is a limited and scarce resource for firms. In keeping with this argument, it shows that the investor attention mediated the relation between environmental penalty and stock market reaction, while the direct effect of environmental penalty on stock market reaction has not been verified, thus, investor attention plays a complete mediating role between them. It claims that significant discrepancy existed in the stock market reaction of enterprises between heavy pollution industries and non-pollution industries, and the results indict that the moderating effect of air pollution and industry saliency are verified.

The results show that the negative impact of environmental penalty on shareholder wealth is not significant in the long term, suggesting that such environmental penalty creates little economic incentive to promote enterprises to increase environmental investment and reduce pollution incident. Therefore, several theoretical contributions are achieved in this study. First, it is fully discussed that the role of investor attention in the impact of environmental penalty on stock market reaction. From attention driven perspective, we assume that media attention becomes investors’ choice set, then the subsequent emotions and preferences are the key to investor attention. Although the statistical results show that the scale of the environmental penalties is insignificant that it is difficult to have a direct impact on their financial performance. However, the most important effect of environmental penalty is to prompt external stakeholders to revise their estimates of the present value of the company. The announcement of environmental punishment may play an important signal role, which expected long-term profitability: a superior environmental performance may indicate a greater ability to increase revenues and generate environmental cost saving. On the contrary, environmental penalties mean reduced revenues and increased costs. Environmental penalty, therefore, is more likely to affect investors’ future expectations of corporate value. Second, based on the expectancy violation theory, companies in heavy pollution industry might experience safety-in-numbers effect which would weaken the directly negative impact of environmental penalties. This is not consistent with the previous impression of heavy pollution industry. It is generally believed that enterprises in heavy polluting industries will suffer more serious negative impacts once they suffer environmental accidents than enterprises in non-heavy polluting industries.

Finally, the findings of this study shed light on how the air pollution moderate on the relationship between the environmental penalty and investor attention. It reveals that higher PM 2.5, the higher air pollution likely to stimulate their perception of the pollution risk, which will amplify the negative effect of environmental penalties on investor attention. Overall, the findings of this study support and extend the view of expectancy violation and attention driven, and improve theoretical support for government to formulate effective environmental strategies.

## 2. Theoretical Foundations and Hypothesis Development

The link between environmental event and stock market reaction is an active research area. As shown in Table 1, based on the review related literature, it is found that the scholars have made contributions to this area. Although the initial majority of studies focused on the developed countries, scholars have recently begun to use samples drawn from some developing countries such as KoreanKorea, Argentina and China [9,11,12,13]

Further, several factors likely affecting the market reaction may originate from firm-specific differences in firm size and market legitimacy [11]. It would have been interesting in exploring the differences of the effect sizes in particular events. It is obvious that negative events also address quite different aspects of even. Therefore, one potential reason for the variation of the study results may refer to the variety of different events such as environmental disclosure, injuries and fatalities of explosions or toxic release in chemical plants, media report contained in these monthly violation lists, firm’s position in the Newsweek Green Ranking, and environmental CSR [12,14,15].

It is also worth mentioning that the length of the event window has been fully discussed by scholars. Generally, ARs represent the difference between expected returns and actual returns, while CARs are assumed to mean the average effect of an event on the firm’s stock price. Furthermore, for the effect sizes calculated from CARs, this work is distinguished between CARs calculated over the traditional event window and CARs calculated over different lengths of event windows. These studies are distinguished among effect sizes derived from short-term, mid-term and longer-term stock market reaction [6,16]. This suggests that the stock market reaction to negative event may have not been subject to changes over time, while the reaction to positive events is relatively insignificant.

Several theoretical arguments have proposed the different manifestations of and mechanisms for the relationship between environmental penalty and stock market reaction. Given that it is necessary for the study to be considered as a multidimensional structure, research should focus also on differences in specific events tested in theoretical explanations. Moreover, general attitudes concerning environmental issues that may affect investors’ reactions to negative events strongly based on the signal theory [13]. While the beneficial effects of positive events resulting from competitive resources or stakeholder theory have recently been taken into consideration due to an increased attention on sustainability issues. For example, the study analyzes the shareholder value effects announcements of environmental performance by examining the market response to two types of environmental performance. The results show that the market reacts positively to announcements of the Corporate Environmental Initiatives (CEIs) through revenue gains and market reacts positively to announcements of environmental Awards and Certifications (EACs) through cost reduction based on signal theory [4]. Finally, the findings suggest that there is asymmetry in the stock market reactions with stronger reactions to negative even than them to positive events. The results believe that this loss is significantly related to the seriousness of the accident. Although the initial short-term and longer-term stock market had a significant positive response to the firm’s position, the deterrence effect of the stock market in the mid-term for environmental problems is weak [11].

However, despite abundant studies that have sought to explore this issue, the cumulative evidence so far is basically inconclusive. The mechanism explanation of this effect is still relatively lacking, further discussion is needed.

### 2.1. Environmental Penalty and Stock Market Reaction

With increasing attention paid to environmental issues, people have higher expectations for environmental management of enterprises [4,17]. Consequently, it becomes more difficult to win better social evaluation from the public and market awards due to environmental strategy. For example, McLaughlin [18] found a significant abnormal return of 0.63% for environmental awards by using announcements from 1985 to 1991. However, scholars [4] revealed that the announcements about environmental charitable donations and ISO 14001 certifications would trigger remarkable positive market reaction. By contrast, voluntary emission reduction would result in an adverse market reaction, also as an environmental performance announcement. It can be seen that the stock market reaction associated with announcements of environmental performance was not always positive, because the public has actually higher environmental expectations for enterprises than ever. Hence, even if enterprises meet the industry standards of environmental protection, they will sometimes be regarded by the public as violating their expectations [19]. Environmental punishment originates from the violation of the public’s implicit social contract, which is “a set of values, beliefs and norms” maintained by the stakeholders [20]. According to these values, believes and norms, they reward or punish a company. Therefore, environmental punishment indicates violation of the society’s expectations of the company’s ability to act, namely, the implied commitment to its appropriate behavior [21,22,23]. The larger environmental penalties due to the greater negative impact caused by the incident, the greater deviation degree from public expectations, the more profound the negative impact on the stock market. Therefore, this is actually the result of external stakeholders voting with their feet.

The environmental penalty will not only incur considerable financial damage for firms, but also tarnish their organizational reputation and image [24,25,26]. Undoubtedly, environmental accidents affect firms’ cash flows [27], increase political costs [28], and bring about reputation penalties [29,30], which could be reflected from the market response. The findings also showed that the market value of companies facing negative events experienced a drop by 0.1% on average [9].

The conceptual framework builds on expectancy violation theory. Specifically, it is expected that the announcement of environmental penalty for environmental evens will help the stakeholders revise and update their expectations and assessment of the firm’s future environmental performance. Using a expectancy violation theory framework, and drawing especially on recent applications of the expectancy view in the environmental management context [31], it is belived that the investor reaction in the stock market is likely related to revised stockholder expectations that the firm would benefit (lose) in terms of higher (lower) expected future shareholder value from more (less) positive stakeholder perceptions of firms’ environmental performance related to the announcement disclosed by the firms.In addition, this issue is discussed from the perspective of externalities. Pigou tax is generally considered to be an effective means to solve the externality problem. By punishing environmental pollution, it can indirectly increase the use cost of resources. It shows that environmental penalties from the government causes the firms to adopt the same or less polluting technologies, aggregate emissions decrease and welfare improves. Meanwhile, if environmental penalties go down, firms will choose more polluting technologies, aggregate emissions will increase and welfare deteriorates [32]. Thus, the hypothesis is proposed:

**Hypothesis** **1** **(H1).** *Environmental penalties have a negative effect on stock market reaction.*

### 2.2. Environmental Penalty and Investor Attention

Investor attention is a limited and scarce resource [33]. Barber and Terrance [17] supposed that individual investors are net buyers of attention-grabbing stocks, which in the news are featured by high abnormal trading volume or high one-day return. It is hard for investors to search for all stock information. Therefore, based on the attention driven theory, we assumed that most investors only consider buying stocks that first attract their attention. These well-known or attention-grabbing stocks tend to receive more attention from investors, and they are often not considered until they can attract investor attention [34]. It is generally considered that there is a significant relationship between media coverage and investor attention. Many investors intend to actively seek information from the media, which can help them increase the certainty of judgment and make them understand the facts more comprehensively [23]. It is supposed that the media only determined the selection set of investor attention [34]. While investor’s attention is an evaluation process, as attention and evaluation are distinct cognitive processes [35]. As a limited resource, more attention is paid to the future returns of the stocks they bought, so investors will consider economic and emotional advantages. Similarly, investor attention is related to the risks they perceive in the information. By collecting risk information of key companies, the information asymmetry in the trading market can be minimized.

Media coverage has an important impact on investor attention, especially on the perception of organizational behavior and internal characteristics. Generally, negative coverage has more extreme influence and attract more attention as well as evaluation than positive news [36]. That is to say, bad news travels fast. Psychologists and management experts believe that media coverage is related to the scale of negative events. The wider scope of negative events, the more likely it will be to attract media coverage, thus causing more public derogation [37]. In this study, the higher environmental penalties faced by firms will damage their reputation and easily attract higher-level media attention and scrutiny [38].

This will seriously affect the stakeholders’ cognition of the enterprise. In their view, the punishment incident reflects the enterprise’s environmental governance strategy, so they will question the enterprise’s ability to create value in the future. Over time, it will also reduce stakeholders’ expectations of enterprise value. This environmental accident is likely to be interpreted as wrongdoing by the media as it violates the expectations of stakeholders for the company’s reputation and commitment. Therefore, it may make stakeholders aware that the company will encounter more environmental risks, violate stakeholders’ expectations and cognition of the company’s behavior, and lose the support of stakeholders.

It is a particularly severe threat exposed by organizational violations, because it predicts the possibility of future illegitimate practices, which may make stakeholders perceive that the company’s behaviors violate their expectations of value consistency. The perception of the severity and controllability of illegal activities has an important impact on the emergence of moral and value standards unanimously accepted by the public. Regular and predictable behaviors are assessed by partners [36], so such illegal behaviors provide strong signals to attract and maintain mainstream attention [39]. The investors’ attentions depended on their perception of information and analysis of value judgment. Judging from additional explanation of this study, the announcement of environmental punishment is not the negative information of firms, but a released risk signal for stakeholders. Therefore, the volatility of stock return and risk premium are the two factors that investors pay most attention to.

In short, the media coverage only determined the selection set of investors, subsequent emotions and preferences become the key to attract investor attention. Based on the theory of limited attention, individual investors are net buyers of eye-catching stocks, so increasing the popularity of firms may attract new investors. However, it is an entirely different story when it comes to the negative news that causes volatility of stock returns and risk premium. As a result, attention can even affect the imbalance among investors who already own shares. The environmental accident that violates the expectations of stakeholders will attract media coverage, which will in turn attract investors that may incorporate the company into their choice set based on their limited attention. In addition, the effects of attention driven are controlled by short-sale on strain. New investors will take a relatively cautious and pessimistic attitude towards such special events with greater volatility of stock returns and risk premium. As a result, the larger amount of an environmental penalty, the more negative media will be attracted. Similarly, more risk signals released by pollution incidents will make investor attention lose its due significance and value. Hence, It is proposed: Similarly, more risk signals from pollution events will distract investors from the meaning and value they deserve.

**Hypothesis** **2** **(H2).** *Environmental penalties have a negative impact on investor attention.*

### 2.3. Mediating Effect of Investor Attention

Based on the data of A-share listed companies in China, the empirical results show that increased investor attention might lead to a remarkable stock market returns over time [40]. Original news headlines, highly abnormal trading volume, and extreme returns could easily attract investor attention [41]. Researchers argue that investors are more inclined to trade stocks with greater attention than those with lower attention, which will obtain relatively higher abnormal returns in the meanwhile.

What investors are most concerned about is not only the future stock returns but also the risk of uncertainty. On this basis, financial scholars suggested that limited investor attention exerts a vital impact on stock market returns because it plays a very critical role in the learning and trading behavior of investors [42,43,44]. Scholars have discussed the prevailing influence of investor attention and learning uncertainty on asset prices [33]. Investor attention is related to stock return variance and risk premium, and this growing relationship is quadratic. Siering [45] finds that media sentiment increased the positive impact on abnormal returns with great investor attention. Researches also show that the volatility of stock return and risk premium increased with investor attention [46,47]. Therefore, great attention will induce higher return volatility. Besides, due to the volatility of returns generated by greater attention, investors need a more substantial risk premium to bear the risks caused by such an attention. On the contrary, lower attention creates lower return volatility, and thus triggers a smaller risk premium.

Based on the investors’ online search behavior exclusively provided by “Google insights for the search”, a model of investor attention was constructed. The results show that investor attention is one of the crucial factors that affected the illiquidity and volatility of the stock market. Moreover, it is closely related to the trading volume of the stock [48]. Scholars take Baidu index as a proxy for investor attention, the results indicate that it has a significantly positive correlation with the stock price that day [49]. Chemmanur and Yan [50] discuss the influence of advertising on both short-term and long-term stock returns. The findings demonstrate that a more substantial stock return caused by attracting investor attention in the advertising year, while a smaller stock return with gradually fading attention in the year subsequent to the advertising year.

As indicated by the above research, investor attention usually leads to higher stock returns, but it may be associated with a greater risk premium as well. Limited investor attention determines that investor attention depend on their preference for higher return or lower risk premium. Thus, the hypothesis is proposed:

**Hypothesis** **3** **(H3).** *Investor attention mediates the relationship between environmental penalties and stock market returns.*

### 2.4. Moderating Role of Air Pollution

The air pollution has become the focus on public attention. Air pollution has now become an important part of weather forecasting. The public concern about the atmospheric environment in which they live also suggests that there is a great concern about the effects of changes in air pollution on their physical and psychological conditions [51]. The situation of the environment will affect human emotions, and to a certain extent, it will interfere with individual behavior [52]. According to previous studies, it is reasonable to assume that air pollution has been linked to psychological distress, especial depression, anxiety, and other mood disorders [53]. For instance, Sass et al. [54] found that PM2.5 has a significant positive impact on negative emotion. Similarly, scholars believe that air pollution, especially the higher PM2.5, is related to negative emotions, such as pessimism and stress. Furthermore, air pollution can also cause emotional fluctuations, which will affect individual performance. For example, the cognitive impairment and negative emotions caused by air pollution will also have a negative impact on individual performance [55]. Even the air pollution can increase unethical behaviors by triggering anxiety [56]. It is well known that emotions play a key role in human social and economic decision-making [57,58]. When the individual emotions are optimistic, their behavior will be radical; when they are pessimistic, their behavior will be conservative. Similarly, emotion do affect the public expectations. The negative emotions also increase individual risk perception, and especially the risk is associated with pollution events [59]. The influence of air pollution on investors’ forecasting behavior in a heterogeneous risk environment is further discussed.

Air pollution changes investors’ risk aversion by affecting investors’ sentiment [60]. With the deterioration of air pollution, investors are more conservative in predicting the prospects of companies suffering environmental penalties. This shows that the behavior of investors is related to the degree of uncertainty they face. The higher uncertainty, the more significant the impact of air pollution on investor attention. This change is even more pronounced in high-risk uncertainty scenarios. Similarly, since air pollution does affect people’s expectations, it affects people’s behavior. It is generally believed that investor attention, as a limited resource, is influenced by investor emotion and preferences. In high air pollution, investors are more likely to stimulate negative emotions, especially when the judgment of corporate value is due to pollution evens. It is easier to stimulate investors’ risk perception and pessimistic expectations than when air pollution is poor, thus reducing investor attention. In the case of high air pollution, investors are more likely to have negative emotions, especially the judgment of enterprise value due to pollution accidents. Compared with the case of good air pollution, it is easier to stimulate investors’ risk perception and pessimistic expectations, thus reducing investor attention. Thus, the hypothesis is proposed:

**Hypothesis** **4** **(H4).** *Air pollution positively moderates the relationship between environmental penalties and investor attention; the higher air pollution, the greater the effect.*

### 2.5. Moderating Role of Industry Saliency

According to the above analysis, the differences in investor attention associated with environmental penalties between heavy pollution industry and non-pollution industry are further discussed. Specifically, while evaluating a company, stakeholders may consider it as a part of the whole industry, rather than as an independent individual. The public usually classifies industries in which organizations are located into different categories, so companies from similar industries are often regarded as following the same organizational rules of professional behavior [61]. As a complex system, an enterprise is different from an individual, in that the negative spillover effects produced by wrongdoing of the others in the whole industry [62]. Stakeholders are more vulnerable to negative stimuli from other companies in the same industry when they make judgments about companies, because the public is likely to pay attention to the negative events exposed by the industry [63,64]. The firms belong to the industry. Therefore, negative events, such as an environmental penalty due to environmental pollution, are more likely to attract the public’s attention [65]. As a complex system, different from an individual, enterprises will suffer more negative spillover effects due to the wrongdoing of other enterprises in the industry [62]. When judging the company, stakeholders are more likely to be negatively stimulated by other companies in the same industry, because the public may notice the negative events exposed in the industry [63,66]. These companies belong to this industry. Thus, negative events, such as fines resulting from environmental pollution, are more likely to attract public attention [65].

In addition, the stereotype of some industries is not only the collective label given by the public, but also the attribution process for the behavior of some enterprises in the industry, which links the industry with the negative evaluation that obviously violates the value judgment of external stakeholders [20].

This categorization causes stakeholders to stereotype the organizations, evaluating them according to the categorical attribute of the industry as a whole, rather than treating it as a distinctive entity [31]. Thus, a stereotyped industry is usually considered to have fundamental defects because its category is associated with relevant negative evaluations. Consequently, relevant enterprises in this industry will also be regarded as violating the external stakeholders’ value judgment and expectations [67]. In such a different industry, a company is often associated with negative stereotypes. Therefore, an organization’s stereotype is possibly due to the negative social evaluations of another organization, which causes stakeholders to “disagree” with the organization and reduce their expectations of organizational ethical behavior [68,69]. Most people believe that an enterprise should protect the natural environment and provide safe and reliable products, which is a fundamental duty that enterprises are supposed to take for granted.

Further, there are some specific enterprises that particularly cause environmental pollution and destruction by their products and production process, thus known as the heavy pollution industry. Zijin Mining incident, the oil spill in the Bohai Sea of ConocoPhillips, and massive water pollution incident in Tuojiang River of Sichuan Province are among the typical cases. All the enterprises that caused the above accidents are from the heavy pollution industry. If a particular industry is novel or unusual in its category, it is more prominent or conspicuous than other industries. Constant exposure of environmental incidents in heavy pollution industry has attracted considerable public attention and thus raised the salience of the industry. Due to frequent major environmental events in heavy pollution industry, an enterprise in that industry will incur disproportionate condemnation and reduce expectations of stakeholders for its environmental protection behavior. Similar reasoning was applied to non-heavy pollution industry. If an individual company in the industry engages in wrongdoing, the pollution incident will be serious because of its scarcity and freshness. In this case, the negative impact on the company far outweighs its actions. Thus, in a heavy pollution industry with a stereotype imposed, there will be the safety-in-numbers effect on the target companies suffering environmental penalties. Lowering public expectations for the industry as a whole will directly decrease the negative impact on the target companies [62,70].

It has been suggested that for the purpose of accurately forecasting the market reaction to different degrees of environmental pollution behavior of a company, it is necessary to explain the expectation level of the public to the whole industry. In the meantime, this effect can also explain the weakening of investors’ perception of risk and the possible stock volatility for the target enterprises in heavy pollution industry. Nevertheless, it will undoubtedly strengthen investors’ perception of risk and the possible stock volatility for the target enterprises in non-heavy pollution industry. Thus, we propose:

**Hypothesis** **5** **(H5).** *Industry saliency moderates the relationship between environmental penalties and investor attention. The investor attention to environmental penalties is negative for firms, and the negative investor attention to environmental penalties of firms in non-pollution industry is more reliable than that of firms in heavy pollution industry.*

In conclusion, whether or not stock market reaction to the environmental penalties of firms remains ultimately an empirical issue. Then a number of hypotheses developed based on theories of expectancy violation and attention driven. We deeply discuss the mediating effect of investor attention on the stock market response to environmental penalties. Furthermore, the moderating effects of the air pollution and industry saliency on environmental penalties to investor attention are discussed. In the next section, we present a number of empirical studies and discuss the results of those hypotheses developed above.

## 3. Methods

**Figure 1:**
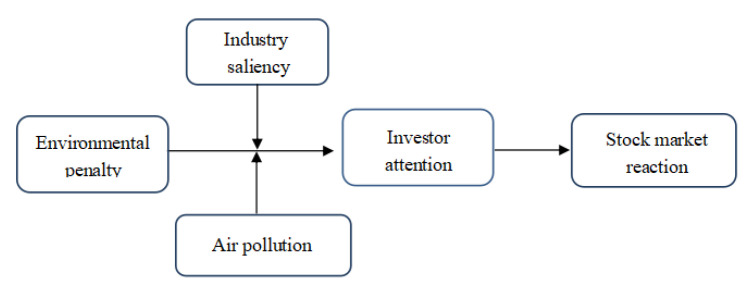
Conceptual model linking environmental penalty and stock market reaction.

To explore the impact mechanism of environmental penalties on stock market reaction, a conceptual model is constructed based on the theories of expectancy violation and attention driven. Figure 1 summarizes the conceptual model of this study.

### 3.1. Sample and Methodology

The response of stock market reaction to environmental penalties is tested through the abnormal returns of A-share listed companies in Shanghai and Shenzhen stock exchanges due to environmental penalties.

To gain insight into environmental penalties of companies, it isutilized the bulletins of the companies on Juchao Network (a network that primarily reports business-related news and is commonly used in literature on the event study). Up to 31 December 2020, data on all environmental incidents during the implementation of the Environmental Protection Law were collected, and then a total of 88 companies were selected as the samples.

A-share listed companies are taken as the research object, because they are required to report financial statements and issue penalty announcements. Due to their popularity in the media, they are more likely to attract the attention of investors, which in turn suffers greater pressure to meet stakeholders’ expectations as well. The statistical data of the sample indicates remarkable diversity in both the number of companies being punished and the amount of the environmental penalties. For the companies that had been punished multiple times, the date and amount of the last punishment are selected as the indicators. Besides, the number of penalties is used as a reference indicator.

To evaluate the abnormal return and stock price regarding a specific event, the influence of the entire market on the stock price must be simultaneously controlled [71]. Abnormal returns are estimates of stock price changes associated with the events. The basis of the event research method is that the influence of events on shareholder wealth is directly reflected in the fluctuation in stock prices in the efficient market. The stock prices of the companies demonstrate that all expected future cash flows are discounted at risk and over time, which are expected to accrue to shareholders. The announcement of listed companies sends such signals as investment quality to consumers and investors [71]. When the announcement signals the positive change in future cash flow, the stock price will rise. On the contrary, when the announcement signals the negative change of future cash flow, the stock price will fall.

Abnormal returns are assessed for four separate time periods. In the first period, the instantaneous effects of companies’ environmental penalties are measured. According to previous event studies, reactions before and after the event are usually estimated and attributed to the event under study [72,73]. In this paper, the stock market reaction is assessed over the 11 trading days because the environmental penalties are gradually unfolded during the period. To explain the ongoing response to the environmental damage incident, we estimate the stock market reaction on the day of the announcement and over the following two weeks of trading days. Calendar days are converted to event days, and the date of the announcement of the environmental penalty is the event day, or Day 0. Day 1 is the first trading day after the event day, and Day-1 was the trading day before the event day. In addition, the event day represents the number of trading days relative to a given event, which is different from the calendar day.

**Figure 2:**
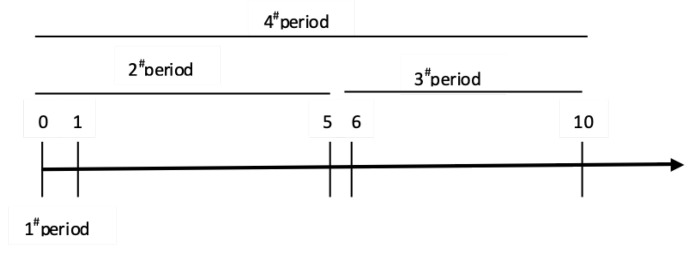
Timeline illustration of event periods.

Figure 2 timeline illustrating the relationship of days and the event day for the four event periods considered. In the first period, the immediate effects of the announcement of a company’s environmental penalty are measured, and the comprehensive stock market reactions on Day 0 and Day 1 are detected. In the 2nd period comprised six trading days to capture the effects of the event day and the first trading week respectively, are regarded as the second period of measurement to investigate the impact of environmental penalties over a long time. The following five days in the second trading week after the event date is viewed as the third period to examine the severity of the longer-term impact on enterprises caused by environmental penalties. In the fourth period, an 1-day period including the 6-day second period and the 5-day third period were used to estimate the impact of environmental penalties on companies. In other words, the aggregate stock market effects were analyzed in the fourth period.

### 3.2. Variables

Based on the above analysis, we find that the mean (−0.65%, −1.727 *), median (−0.49%, −2.006 *), and percent of negative abnormal returns (40.90%, −1.599 **) on Day 1 are negative and significant. The abnormal returns on Day 1 are taken as a subject. Environmental penalty. To further reflect the impact of environmental penalty on the financial performance of a company and avoid the impact of differences between samples on the research results to the greatest extent, the ratio of the environmental penalty amount to the net profit in the last quarter are taken as an independent variable [2,74].

Investor attention.According to previous research, “Google insight search” or “Baidu index” have been frequently used as the proxy variable of investment attention [75,76]. Accordingly, investor attention in this paper is built based on Baidu index which could reflect investors’ online behavior. Therefore, in view of the research background of this paper, we take A-share listed companies as a sample and use the Baidu Index of related companies as the measurement of investor attention.

(1) $$ABI_{it}=\left(BI_{it}-\hat{B}I_{it}\right)/\hat{B}I_{it}$$

where $ABI_{it}$, $BI_{it}$ and $\hat{B}I_{it}$ denoted abnormal Baidu index, daily Baidu index, and expected Baidu index respectively of stock I on Day 0. The daily Baidu index is the Baidu index of stock I on Day 0. The expected Baidu index $\hat{B}I_{it}$ is obtained by time series calculation.

Noteworthy, the event analysis method mainly tests the impact of a special event on the stock market reaction of an enterprise, thus implying that the trend of previous attention as well as the “abnormal returns” should be considered. Thus, we measure investor attention as the abnormal Baidu index. Firstly, we gather the Baidu index on the Internet from 2006 to the event date with the names of companies as the keywords. For example, in the case of Shanghai Jinghua Adhesive New Materials Co., LTD., Baidu index tack “Shanghai Jinghua” as the keyword. Secondly, the time series is created by Baidu index within two to three years before Day 0, and autoregression is carried out to obtain the predicted value of Day 0. Finally, according to the measured data, abnormal Baidu index of Day 0 is calculated.

Noteworthy, the event analysis method mainly tests the impact of a special event on the stock market reaction of an enterprise, thus implying that the trend of previous attention as well as the “abnormal returns” should be considered. Thus, we measure investor attention as the abnormal Baidu index. Firstly, we gather the Baidu index on the Internet from 2006 to the event date with the names of companies as the keywords. For example, in the case of Shanghai Jinghua Adhesive New Materials Co., LTD., Baidu index tack “Shanghai Jinghua” as the keyword. Secondly, the time series is created by Baidu index within two to three years before Day 0, and autoregression is carried out to obtain the predicted value of Day 0. Finally, according to the measured data, abnormal Baidu index of Day 0 is calculated. Stock market reaction. The dependent variable is based on CAR (cumulative abnormal return) on Day 1.

(2) $$AR_{it}=R_{it}-{\hat{R}}_{it}$$

where *A*$R_{it}$, $R_{it}$, anddenoted abnormal return, daily return, and expected daily return respectively of stock I on Day t. The expected daily return ${\hat{R}}_{it}$ is calculated according to the market model in the following equation:

(3) $${\hat{R}}_{it}={\hat{\alpha }}_{i}+{\hat{\beta }}_{i}R_{mt}+{\hat{\varepsilon }}_{it}$$

where $R_{mt}$ is the whole stock market return on Day t. ${\hat{\beta }}_{i}$ and ${\hat{\beta }}_{i}$ can be estimated over a period of 200 days starting from 120 days prior to an event and up to 30 days prior to the event.

To compute each sample firm’s expected return, $a_{i}$ and $b_{i}$ are estimated using ordinary least squares regression over the estimation period of 200 trading days. In accordance with many event studies, we begin the estimation period 11 trading days prior to the announcement day of environmental penalties, and end it 11 trading days later.

CAR was calculated as follows:

(4) $$CAR_{\left(t2,t1\right)}=\sum_{t1}^{t2}AR_{t}$$

where $t_{1}$ and $t_{2}$ denoted the beginning and the end of the event window, t[(t1, t2)].

Air pollution. Relative rates of PM 2.5 are taken as the measure of variables, the average PM 2.5 in a week before Day 0 is also taken as the base to calculate the relative rate of PM 2.5 every day. We tack the mean value of PM 2.5 in seven Chinese cities (including Beijing, Shanghai, Tianjin, Chongqing, Guangzhou, Nanjing and Hangzhou) as index, the data of which comes from Air Quality Index published by the Ministry of environmental Protection of China.

Control variables. Because larger firms may not only attract media attention, but also attract investor attention. Thus, we control for age and size using logged measures of both years and total sales. We control for ownership type by adding a dummy coded 1 for state-owned listed companies. Because high-performing organizations may be shielded from environmental penalties, we control for performance as measured by sales and net profit. Finally, we control for the effect of punishment imprinting by checking if the firm has been punished.

## 4. Results

### 4.1. Stock Market Reaction to Environmental Penalty

Table 2 presents the descriptive statistics for the sample of the 88 firms. It indicates that the mean (median) total assets are $981.830 million ($495.163 million), sales are $590.385 million ($212.078 million) and the mean (median) net profits is $5.496 million ($1.910 million).

Table 3 displays the results for the sample of the 88 companies. It indicates that on the event date Day 0 and over the 10 following trading days, abnormal returns are related to environmental penalties. Considering the relatively small sample of this study, the median and percentage negative (positive) abnormal returns are more appropriate than the mean abnormal returns in terms of reflecting the fluctuation in abnormal returns of the sample. The median abnormal returns on Day 1, Day 2, Day 3 and Day 4 are −0.650%, 0.59%, −0.939% and −0.881% respectively, which are negative and similar, and statistically significant at the 5% and 10% level. The mean abnormal returns on the Day 1, Day 3 and Day 4 are −0.49%, −0.937% and −0.908%, which are significantly negative at the 5% and 10% level. Furthermore, the percent negative abnormal returns during Day 1 to Day 5 are substantially different from 50%, 36 out of the 88 firms (40.90% of the sample) and 52 with out of the 88 firms (59.09% of the sample) at the 10% level.

During the 6-day period from Day 0 to Day 5, the results show that the median and mean CAR are −3.12% and −4.03%, which are significantly different from zero at the 5% level. The percent negative CARs are constantly insignificant during the 6-day period from Day 0 to Day 5, which is 52 with out of the 88 firms (60.47% of the sample) at the 10% level.

To summarize, although there is a modest and significant negative stock market reaction to environmental penalties, the median, mean, and the percent negative of abnormal returns are negative and significant on Day 1, Day 3 and Day 4. Similarly, there is no significant response to CARs in discrete periods, except for the 6-day period from Day 0 to Day 5.

Abnormal returns for the sample of 59 firms in heavy pollution industry and 29 firms in non-heavy pollution industry that are penalized because of environmental pollution. Event Day 0 denote the date of the announcement of environmental penalties. Panel A of Table 4 present the descriptive statistics for the sample of 59 firms in heavy pollution industry. It indicates that the mean (median) sales are $648.140 million ($278.028 million) and the mean (median) net profit is $35.004 million ($15.608 million). Panel B of Table 4 present the descriptive statistics for the sample of 24 firms in non-heavy pollution industry. It indicates that the mean (median) sales are $388.933 million ($153.346 million) and the mean (median) net profit is $14.362 million ($6.004 million). Furthermore, our results show that the mean (median) penalty amount in heavy pollution industry is CNY 0.406(0.200) million and the mean (median) penalty amount in non-heavy pollution industry is $1.747 million ($0.41 million).

Based on the Panel A and Panel B of Table 4, the comprehensive strength, as reflected from the sales and net profit of enterprises in heavy pollution industry is more competitive than that in non-heavy pollution industry. However, the mean and median of penalty amount in heavy pollution industry are smaller than those in non-heavy pollution industry. The mean penalty amount of non-heavy pollution industry is much higher than that in heavy pollution industry, because the penalty amount of Chongqing Beer is up to $1.863 million, far exceeding the average level of the industry. Similarly, this also verifies that the median is closer to the real situation of small samples.

Abnormal returns between enterprises in the heavy pollution industry and non-heavy pollution industry differed, and Day 0 is the day when the environmental penalties are announced. Table 5 presents the stock market reaction associated with different industries. As reflects in Panel A of Table 5, in terms of companies in heavy pollution industry, only the median and mean abnormal return on Day 1, being −0.799% and −0.479%, are distinguished from zero at a statistically significant level of 10%. And 37 out of the 59 firms (62.71% of the sample) experience negative abnormal returns on Day 1, which are distinguished from 50% at a statistically significant level of 10%. On the other hand, the data from Panel B shows that for enterprises in non-heavy pollution industry, the median and mean abnormal returns on Day 1 are negative and significant, being −0.33% and −1.26% respectively. In addition, the median and mean abnormal returns on Day 2 are −0.92% and −1.595%. Furthermore, panel A displays that the median abnormal returns on Day 4, Day 9 and Day 10 are negative and significant, being −0.903%, −1514 and −1.110% respectively, and the mean abnormal returns on Day 4 and Day 10 also are negative and significant, being −0.984% and −2.235%. Panel B indicates that none of the median and mean abnormal returns is significant.

Panel A shows that the mean CAR during the 5-day period of Day 6 and Day 10 is −6.564%, which is significantly different from zero at the level of 10%. In addition, the median and mean CARs during the 11-day period of Day 0 and Day 10 are −9.948% and −9.332%, which is significantly different from zero at the level of 10%. While Panel B also displays that the mean CAR during the 2-day period of Day 0 and Day 1 was −1.110%, which is significantly different from zero at the level of 10%. However, no other mean CARs were significantly different from zero in non-heavy pollution industry.

To sum up, although there was a modest and significantly negative stock market reaction over the first trading period after the announcement of environmental penalties in all industries, the stock market respond of heavy pollution industries and non-heavy pollution industries is significantly different. For non-pollution industries, such magnitude and significance of the negative reactions of stock market have not dissipated in the following period after Day 0, while for heavy pollution industries, the long-term of the negative reactions of stock market reaction on Day 9 and Day 10 are still significant.

### 4.2. Hypothesis Testing

Table 6 displays the descriptive statistics and correlations between the variables examined. The results demonstrate a significant negative correlation between air pollution and abnormal return, thus supporting the contention that they are distinct constructs and being consistent with prior research [77]. The results also reveal that there is a strong negative correlation between the environmental penalty, investor attention and industry saliency, while there is a strong positive correlation between investor attention and abnormal returns. The punished numbers are positively correlated with the ownership type and environmental penalty, but negatively correlated with investor attention.

According to Table 7, Model 1 included the focal predictor variable, the environmental penalty, and the control variables. The results show that total assets are negatively and significantly related to abnormal return (β = −0.318, *p* < 0.1), while sales (β = 0.287, *p* < 0.1) and ownership type (β = 0.248, *p* < 0.05) are positively and significantly related to abnormal return. However, the environmental penalty is negatively but insignificantly related to abnormal return (β = −0.120, *p* > 0.1), thus failing to support H1.

Model 2 included the focal predictor variables, environmental penalty, investor attention and control variables. The results claim that the environmental penalty is negatively but insignificantly related to abnormal returns (β = −0.068, *p* > 0.1), then investor attention is positively and significantly related to abnormal return (β = 0.214, *p* < 0.1). Model 3 also included the focal predictor variables, environmental penalty and control variables. The results indicate that the environmental penalty is negatively and significantly related to investor attention (β = −0.279, *p* < 0.05), thus supporting H2.

H3 discusses the mediating role of investor attention in the relationship between environmental penalty and stock market reaction. The results indicate that environmental penalty is negative and significant effect on investor attention (β = −0.279, *p* < 0.005). Based on the above results, H3 fail to be completely verified, thus requiring further exploration. To further test H3, the effect of investor attention needs to be investigated. Consequently, 10,000 samples under 90% confidence interval are selected and bootstrap is used to test the mediating variable. In this model, the results show that in the case of the whole sample, (β = 0.0163, SE = 0.0077), (CI: 0.036, 0.291), zero was excluded. Therefore, investor attention plays a significant mediating role in the relationship between environmental penalties and abnormal returns, supporting H3.

**Figure 3:**
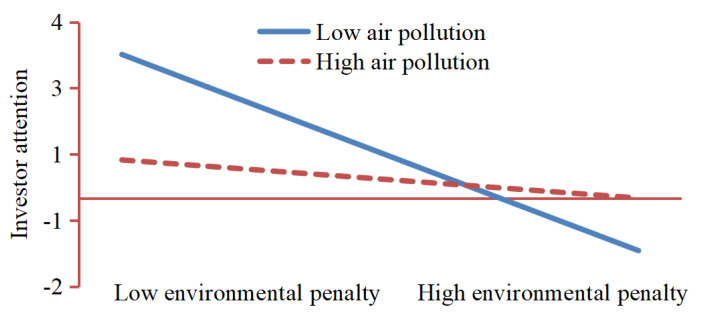
Moderation effect of air pollution on the relationship between environmental penalty and investor attention.

H4 predicts air pollution will attenuate the negative effect of environmental penalties on investor attention. We find that the environmental penalty is negatively and significantly related to investor attention (β = −0.243, *p* < 0.05), while air pollution is negatively but insignificantly related to investor attention (β = −0.002, *p* > 0.1) in model 4. Model 5 confirms that the interaction term of environmental penalty and air pollution is positive and significant (β = 0.274, *p* < 0.1), but the air pollution is negative and insignificant (β = −0.014, *p* > 0.1). Thus, H4 is supported. Figure 3 illustrate these relationships, showing that environmental penalty is more effective with increased levels of air pollution.

**Figure 4:**
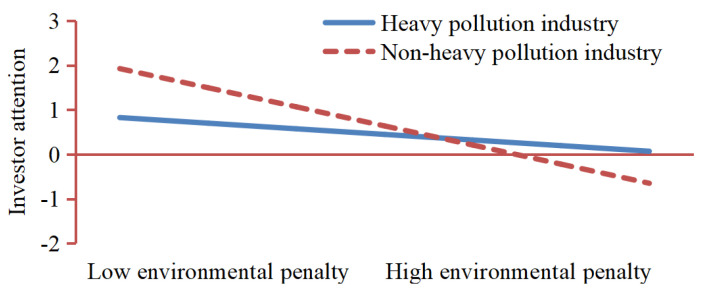
Moderation effect of industry saliency on the relationship between environmental penalty and investor attention.

H5 predicts a moderating effect of industry saliency on the relationship between environmental penalties and investor attention. The results manifest that the interaction term of environmental penalty and industry saliency was negative and significant (β = −0.184, *p* < 0.1). We firstly test two sets of samples respectively. According to the industry standard, the sample is divided into two groups. One group included the 59 listed companies from heavy pollution industry, and the other included the 29 listed companies from non-heavy pollution industry. Secondly, the two groups are verified successively. The main purpose is to test the difference between the two groups in the impact of environmental penalty on investor attention. Finally, the regression results show that the environmental penalty in non-heavy pollution industry is negative and significant (β = −0.429, *p* < 0.05). In addition, the regression results indicate that environmental penalty in heavy pollution industry is negative and significant (β = −0.126, *p* < 0.1). Figure 4 illustrates that the impact of environmental penalties on investor attention between heavy pollution industry and non-heavy pollution industry differed substantially, and the moderating effect of industry saliency is significant, so H5 is supported.

According to Table 8, a further comparative analysis is made of enterprises suffering environmental penalties in heavy pollution industry and non-heavy pollution industry. The results manifest that both the median and mean of the environmental penalty of enterprises in non-heavy pollution industry are significantly higher than those in heavy pollution industry.

On the other hand, we further analyze the enterprises in non-heavy pollution industry and find that most of them are in a medium or low status in the industry. Thus, according to the characteristics of the two different industries, such pollution incidents in non-heavy pollution industry are mainly attributed to poor management of enterprises. Generally, such an enterprise would not have particularly competitiveness. Similarly, the enterprises with net profit loss in the current month accounted for 20.7% of the sample 29 enterprises. On the other hand, for the enterprises in heavy pollution industry, the results are quite different. The enterprises show a relatively uniform distribution of positions in their respective industries, and those with net profits loss in current month account for 6.78% of the 59 sample enterprises.

In the meanwhile, the difference of investor attention between heavy pollution industry and non-heavy pollution industry is also analyzed. The results demonstrate that the investor attentions in heavy pollution industry on Day 0 and Day 1 are significant and negative in heavy polluting industry and non-heavy polluting industry, while the investor attention on Day 1 was significantly higher than it on Day 0 in non-heavy pollution industry. There are obvious differences between heavy pollution industries and non-heavy pollution industries in terms of both the penalty amount and investor attention. Based on the above results, it could be concluded that for companies in non-pollution industries, the environmental penalty will rise with the increase in the severity of the pollution incident, and it will also violate the stakeholders’ expectations to a greater extent and release more risk signals. Therefore, based on the theory of attention driven, it will lead to a decline in investor attention, and the results just confirmed it. However, enterprises for in heavy pollution industries, the effect of environmental penalty on investor attention is not significantly, which is consistent with our theoretical assumptions based on the theory of expectancy violation. In the case of lower public expectations, an invariable environmental penalty is assumed, and thus it will bring less volatility of investor attention.

## 5. Discussions

### 5.1. Analysis of CAR

**Figure 5:**
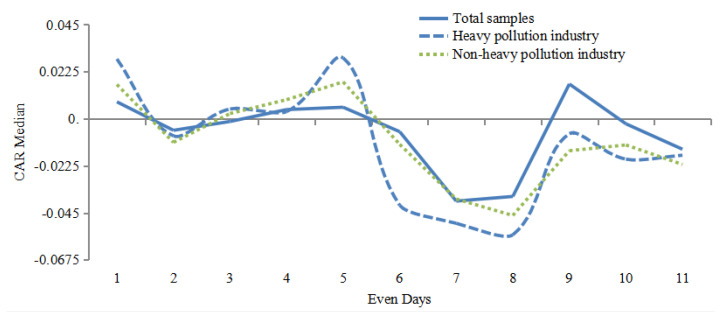
Median CARs for the sample.

Although the CARs of all the sample enterprises from heavy pollution industry and non-heavy pollution industry was negative in most trading periods, it is negative and significant only on Day 2 for non-heavy pollution industry, while it is negative and significant on Day 1, Day 4, Day 9 and Day 10 for heavy pollution industry. The results reflect that the CARs have no particularly strong negative response to environmental penalties, and only slightly fluctuate immediately after the event day. As shown in the Figure 5, environmental penalty would exert negative impact on stock market reaction. H1 is not verified, then the environmental penalties do not directly affect abnormal returns.

It could be seen from the Figure 5 that the fluctuation of CARs in the whole sample is always between heavy pollution industry and non-heavy pollution industry. It also shown that the negative fluctuation of heavy pollution industry is actually weaker than that of non-heavy pollution industry, which also reflects the differences of stock market reaction in different industries. This also served as an intuitive evidence for the moderating effect of industry saliency.

### 5.2. Conclusions

This study explores the impact of environmental penalty on stock market reaction associated with the announcement of environmental punishment. The results show that the scale and significance of the stock market reaction were beyond our expectation. The abnormal returns were negative and significant only on Day 1, and then it gradually dissipated from the following day after the announcement of the environmental penalty. The results also manifest that environmental penalty exert negative impact on abnormal returns. However, the impact of the environmental penalty on shareholder value is not direct and significant. This is in line with our practical experience. Environmental penalties imposed by government departments are so insignificant for listed companies that they all emphasize in their announcements without exception that such penalties have little impact on their net profits. Some scholars also find that the average reduction in estimated market value was far lower than the estimated changes in market value for similar events in other countries, indicating that the negative environmental events of Chinese listed companies had a weak impact on the stock market [9].

Firstly, based on the theory of attention driven, the results indicate that investor attention plays a key mediating role in the relationship between environmental penalty and stock market reaction, while the direct effect of environmental penalties on abnormal returns are not significant, thus which fully reveals that investor attention plays a complete mediating role. Secondly, further analysis reveals that there are still a significantly differences in stock market response between heavy pollution industry and non-heavy pollution industry, and the enterprises in heavy pollution industry may weaken the direct negative impact of pollution incidents due to the safety-in-numbers effect, then the moderating effect of industry saliency is significant. Finallythe sample firms in heavy pollution industry and non-heavy, the moderating effect of air pollution in the relationship between environmental penalty and investor attention has also been verified.

### 5.3. Theory Contributions

Environmental issues have been gaining momentum throughout the last decades in both academic research and business practice. However, most scholars pointed out the lack of appropriate monitoring activities and weak enforcement pertaining to the implementation of environmental regulations. Then there are several theoretical contributions worth highlighting in this study.

First, it is fully discussed the important role of investor attention in the impact of environmental penalty on stock market reaction. Although previous research provided a reasonable explanation for the event analysis study based on signal theory, events will affect the stock market reaction by damaging their organizational reputation and image [73,78], the investor attention is surprisingly absent from the theoretical literature as a force to be reckoned with. It is commonly known that stock markets are not information perfect, they are considered to work with a high degree of efficiency and accuracy by continually assessing and valuing new information [79,80]. Given the small scale of these penalties, it is difficult to have a direct and significant impact on their current cash flow. Most importantly, markets are more likely to revise their estimates of the present value of businesses.

The announcement of environmental punishment can be considered a public signal to a firm’s poor environmental strategy. The environmental penalty can expect long-term profitability as its signal: a superior environmental performance may indicate a greater ability to increase revenues and generate environmental cost saving. On the contrary, a larger amount of environmental penalty indicates a greater environmental risk and a lower shareholder value. So, based on the theory of attention driven, media coverage as the third party will affect investor attention, and thus facilitate their establishment of a selection set, then the subsequent emotions and preferences become the key to investor attention, which are also a meaningful explanation of the impact of environmental penalty on stock market reaction.

The second contribution of this study is that it promotes a more realistic view on the different impact of environmental penalty on enterprises between heavy pollution industry and non-heavy pollution industry. A general assumption is that once enterprises in heavy pollution industries are punished for environmental pollution, they will suffer from more considerable financial and reputation damage than other companies, but this is rarely the case, so individuals need more information to understand the difference. Furthermore, based on the theory of expectancy violation, companies in heavy pollution industry might experience the safety-in-numbers effect which would weaken the directly negative impact of pollution incidents. Due to the stereotype of the heavy pollution industry as a whole, the expectation for the organization to abide by environmental rules has been greatly reduced, and stakeholders’ expectation has also declined seriously. Based on this, we further explain the impact of environmental penalties on enterprises in different industries.

Our final contribution is to test the impact of the key external environmental factors air pollution on the relationship between the investor attention and environmental penalty. Consistent with our expectations, we find that the higher air pollution, likely due to their greater perception of environmental risk, have a significantly stronger negative reaction of investor attention to the environmental penalty. However, contrary to expectations and earlier findings by Lyon and Shimshack [81], it shows that firms with superior financial performance have a significantly stronger negative reaction to their rankings.

### 5.4. Practical Implications

Although the latest Environmental Protection Law is known as the most stringent environmental law in the history, It is commonly believedthat improving the environment should not merely rely on the supervision of the government. In 2018, civil penalties totaled 15,280 million yuan imposed in 186,000 cases, for an average penalty of 821,500 yuan, an increase of 124% and 382% respectively compared with 2014 before the promulgation of the new Environmental Protection Law of the People’s Republic of China. Instead, it requires the joint efforts of all stakeholders, including government inspection, effective supervision from media and non-governmental organizations (NGOs), manufacturing enterprises, and relevant upstream and downstream companies [82]. How to effectively reduce pollution has always been a problem of concern. At present, there are no satisfactory solutions or enforcement activities for enterprises to undertake the responsibility for reducing violations and emissions. Enterprises are the principal part of the market economy, so the most effective way of environmental protection is to give full play to the leverage role of the market. It will promote enterprises to spontaneously improve their technology, improve their production conditions and increase green investment to meet the environmental requirements [83]. Meanwhile, more and more enterprises begin to pay attention to green innovation and green supply chain [84,85].

The current study has provided important practical implications concerning environmental management strategies of enterprises. We believe that our results are consistent with the attention driven theory perspective that we develop in detail: investors attracted to better firms anticipate larger future cash flows due to more positive environmental performance. These benefits would be expected to result, for example, from retention and attraction of superior employees, gains in new business from customers with green preferences, lessening of costly resistance from NGOs. Consequently, firms with superior environmental performance will benefit from more positive stockholder reactions.

Our findings may have a broader impact on environmental protection strategies of the government. With the increasing public awareness of environmental protection, it is particularly important to formulate effective strategies for tackling environmental pollution [86]. The research has also shown that market participants would respond to media reports, but they have not reaction to corporate press releases or to NGOs’ disclosures [82]. From the perspective of organizational evaluation, managers should make full use of the efforts of media, which will attract more public attention and increase the stakeholders’ risk perception, and thereby make them vote with their feet and affect the company’s stock market reaction.

This study demonstrates that merely relying on the power of the capital market is not enough to prevent pollution events, or to encourage enterprises to improve environmental investment. That is, capital markets alone are not sufficient to prevent pollution incidents or to motivate companies to improve their environmental investments. The empirical analysis of Spanish firms has revealed that the input and output of environmental management as two sides of the same coin must be fully considered [74]. As a social citizen, an enterprise should not only contribute its economic strength but also consider the obvious social benefits. In addition to improving its financial contribution, managers should weigh their social responsibility and moral obligation. There is no doubt that firms are also affected by the required changes in the involvement of non-market forces. The government and NGOs should take active part and coordinate the joint efforts of all parties.

### 5.5. Limitations and Further Direction

The limitation of the present research lies in its sample. Although the event study methodology has been widely employed to study the impacts of events on shareholder value, its application is always limited to publicly traded companies. Due to the inherent noise from the stock market, it is difficult to make an accurate evaluation. On the other hand, the sample of this study only consisted of 88 listed companies, which would greatly limit the generalizability and applicability of the research results. Besides, this study has taken Baidu index as the proxy for the changes in investor attention. However, as a comprehensive index, it could not completely and accurately reflect the changes in investors’ attention.

A meaningful direction for future research is to find a better method for calculating shareholder value and to explore alternative proxy variables that can reflect investor attention more accurately. This will promote our understanding of the influence mechanism of environmental penalties on abnormal returns. Furthermore, it will be interesting to explore why the direct impact of the environmental penalties on abnormal returns is not significant and whether such a money punishment can play a substantial role for the listed companies with special competitive strength.

## Figures and Tables

**Table 1:** Studies on environmental events and stock market reaction.

Study	Research Setting	Applied Theory	Event	Modera-tor	Media-tor	Dependent Variable	Hypothesized Relationship	Main Findings
Garner& Lacina (2019)	USA oil and gas firms (2010)	Signal theory	British Petroleum oil spill; Deepwater explosion; President Obama’s drilling ban	-	-	Stock market reaction	Environmental disclosure->CAR (-)	Environmental disclosure ->stock market reaction (-)
Capelle-Blancard & Laguna (2010)	64 explosions in chemical plants worldwide (1990–2005)	Event study	Injuries and fatalities; Toxic release	-	-	Abnormal returns; shareholder loss	Total number of fatalities and serious injuries; released toxic chemicals-> CAR; shareholder loss(-)	The fatality or serious injury is associated with an additional loss. The toxic release is associated with higher losses in longer event windows.
Jacobs et al. (2010)	417 CEI announcements and 363 EAC announcements	Signal theory	Corporate Environmental Initiatives; Environmental Awards and Certifications	Revenue gains; Cost reduction	-	Abnormal returns	CEIs and EACs->the market reaction (+); The market reaction to EACs is greater than that for CEIs	Announcements of philanthropic gifts for environmental causes are associated with significant positive market reaction, voluntary emission reductions are associated with significant negative market reaction, and ISO 14001 certifications are associated with significant positive market reaction.
Xu et al. (2012)	57 listed companies disclosed for environmental pollution in China (2010)	Event study	Pollution type; Disclosure source; Disclosure level; Modernization level; Major shareholder holding level; Company attribute	-	-	Abnormal returns	Pollution type; Disclosure source; level; Modernization level; Major shareholder holding level; Company attribute->CAR(-)	The negative environmental events of Chinese listed companies currently have weak impact on the stock market
Dasgupta et al. (2012)	Korean public disclosure program	Event study	The media report contained in these monthly violation lists.	-	-	Abnormal returns	Environmental news->CAR(-)	The average reduction in market value is higher than them in other countries. The extent of media coverage is positive influence reduction in market value.
Cordeiro &Tewari (2015)	500 firms ranked by Newsweek US 2009	Stakeholder theory	Firm’s position in the Newsweek Green Ranking	-	Firm size; firm legitimacy	Short-term and long-term stock market returns	Firm’s position in the Newsweek Green Ranking-> short-term and longer-term stock market reaction (+) firm’s industry-adjusted ranking based on its Green Score within the industry-> short-term and longer-term stock market reaction (+); firm size and organizational legitimacy as a moderator of investor reaction to environmental disclosure	Stock market investors react positively in terms of both the short and intermediate term; industry-adjusted rankings of environmental CSR and that the investor reaction is significantly influenced by firm size and firm legitimacy
Flammer & Caroline (2013)	Announcement of corporate news related to environment for all USA publicly traded companies (1980- 2009)	Environment-as-a-resource	Environmental CSR	-	-	Stock market reaction; CARs	The announcement of eco-friendly corporate initiatives-> shareholders react (+); the announcement of eco- harmful corporate initiatives-> shareholders react (-); the announcement of eco-harmful corporate events over time-> shareholders’ negative reaction (+); the announcement of eco-friendly corporate events over time-> positive shareholders’ reaction (-)	The negative stock market reaction to eco-harmful behavior has increased, while the positive reaction to eco-friendly initiatives has decreased; The positive (negative) stock market reaction to eco-friendly (-harmful) events is smaller for companies with higher levels of environmental CSR the negative stock market reaction to eco-harmful behavior has increased, while the positive reaction to eco-friendly initiatives has decreased
Grand& D’Elia (2012)	News appearing in Argentine newspapper La Nación (1995–2001) (2003–2008)	Signal theory	Positive environmental news; negative environmental news	-	-	AARs	ARit > 0 for positive announcements; ARit < 0 for negative events	The environmental news can cause impacts on stock returns in developing countries as high as those in developed ones.
Lanoie et al. (1998)	Firms on British Columbia’s lists of polluters American and Canadian	SIMM (single-index market model)	Release of information	-	-	Average abnormal return AAR	Large polluters are affected more significantly by such release than smaller polluters	The capital markets react to the release of information in large polluters are affected more significantly from such release than smaller polluters
Carpentier & Suret (2015)	161major accidents reported on the front page of the New York Times from (1959 – 2010)	-	Accident announcement	-	-	Average compounded abnormal return	Major accident announcement -> market value in the mid-term (-); The negative mid-term effect of an accident announcement on the firm’s market value is lower for environmental than for non-environmental accidents; the negative mid-term effect following an accident announcement on the firm’s market value is stronger for airline accidents than for non-airline accidents; The negative mid-term effect following an accident announcement on market value is stronger for accidents followed by government intervention	The deterrence effect of the stock market in the mid-term for environmental problems is weak
Konar & Cohen (1997)	Firms with TRI emissions USA 1988–1990 and 1991–1992	Market-based incentive	Information on toxic chemical emissions	-	-	Abnormal returns	Market reacted more to unexpected TRI disclosures than to those that were already expected	Firms with the largest stock price decline on the day this information became public subsequently reduced emissions more than their industry peers

**Table 2:** Descriptive statistics for the whole sample.

	Age	Total Assets	Sales	Net Profit	Employees	Penalty Amount
	Year	$million	$million	$million	s	$million
Mean	19.506	981.830	590.385	5.496	3681	0.1324
Median	19	495.163	212.078	1.910	2576	0.2
S.D.	4.552	170.484	0.523	0.523	350.15	0.7261

**Table 3:** Abnormal returns for the whole sample of 88 firms that were penalized for environmental pollution.

Event Days ()	*N*	Median	*Z* ^a^	Mean	*t*	% Negative	*Z* ^b^
0	88	−1.00%	−1.599	0.12%	−0.409	58.00%	−0.428
1	88	−0.65%	−1.727 *	−0.49%	−2.006 *	40.90%	−1.599 *
2	88	−0.60%	−1.831 *	−0.68%	−1.491	59.09%	−1.599 *
3	88	−0.94%	−2.064 **	−0.94%	−1.872 *	59.09%	−1.599 *
4	88	−0.88%	−2.288 **	−1.14%	−2.229 **	59.09%	−1.599 *
5	88	−1.30%	−1.639	−0.91%	−1.542	59.09%	−1.599 *
6	88	−0.65%	−1.153	−0.53%	−0.8	56.82%	−1.173
7	88	−0.97%	−1.277	−0.48%	−0.604	56.82%	−1.173
8	88	−1.30%	−1.419	−0.64%	−0.713	57.95%	−1.386
9	88	−1.51%	−1.897	−1.35%	−1.309	53.41%	−0.533
10	88	−1.16%	−1.964 *	−1.70%	−1.714 *	55.68%	−0.959
(0,1)	88	−0.65%	−1.582	−0.62%	−1.265	56.81%	−1.173
(0,5)	88	−3.12%	−2.064 **	−4.03%	−1.769 **	60.47%	−2.025 *
(6,10)	88	−6.98%	−1.739	−4.69%	−1.133	55.68%	−0.959
(0,10)	88	−2.46%	−1.943 *	−8.73%	−1.465	59.09%	−1.599

** *p* ≤ 0.05; * *p* ≤ 0.10. ^a^ Z-statistics for medians were obtained using Wilcoxon signed-rank tests. ^b^ Z-statistics for % negatives were obtained using binomial sign tests. Note: Event Day 0 denoted the date of the announcement of environmental penalties.

**Table 4:** Descriptive statistics for the sample firms in heavy pollution industry and non-heavy pollution industry Panel A. Heavy pollution industry.

	Age	Market Value	Total Assets	Sales	Net Profit	Employees	Penalty Amount
	year	$million	$million	$million	$million	s	$million
Mean	18.581	1499.3	1532.744	648.140	35.004	3887.419	0.061
Median	18	1061.147	774.5282	278.028	15.608	2943	0.030
S.D.	5.02	1302.967	2170.029	1064.731	56.129	3504.478	0.074
Panel A. Heavy pollution industry							
Mean	19.5	1123.231	823.962	388.933	14.362	3525.292	1.747
Median	19.5	722.024	706.288	153.346	6.004	2335.5	0.041
S.D.	3.833	1292.624	762.037	570.632	32.898	3245.003	7.917
Panel B. Non-heavy pollution industry							

**Table 5:** Stock market reaction for the sample firms in heavy pollution industry and non-heavy pollution industry.

Event Days ()	N	Median	Z ^**a**^	Mean	t	% Negative	Z ^**b**^
0	59	−0.10%	−0.523	0.47%	1.267	54.23%	−0.657
1	59	−0.80%	−1.774 *	−0.48%	−1.209 *	62.71%	−1.823 *
2	59	−0.50%	−0.966	−0.23%	−0.415	59.32%	−1.302
3	59	−0.90%	−1.532	−0.73%	−1.295	59.32%	−1.302
4	59	−0.90%	−1.940 **	−0.98%	−1.776 *	59.32%	−1.302
5	59	−0.78%	−1.004	−0.82%	−1.28	55.93%	−0.781
6	59	0.49%	−0.589	−0.47%	−0.757	55.93%	−0.781
7	59	−0.84%	−0.981	−0.78%	−1.154	57.62%	−1.042
8	59	−1.24%	−1.585	−1.19%	−1.664	59.32%	−1.302
9	59	−1.51%	−1.897 *	−1.96%	−2.049	54.24%	−0.521
10	59	−1.11%	−1.985 **	−2.17%	−2.235 **	57.62%	−1.042
(0,1)	59	−1.04%	−0.823	−0.01%	−0.007	59.32%	−1.302
(0,5)	59	−3.12%	−2.768	−2.77%	−1.085	62.71%	1.823 *
(6,10)	59	−6.10%	−1.623	−6.56%	−1.827 *	54.24%	−0.521
(0,10)	59	−9.95%	−1.744 *	−9.33%	−1.681 *	59.32%	−1.302
Panel A. Heavy pollution industry							
0	29	−0.14%	−1.435	−0.60%	−1.399	58.62%	0.945
1	29	−0.33%	−1.829 **	−1.26%	−1.871 *	58.33%	−0.743
2	29	−0.92%	−1.762 *	−1.60%	−2.002 *	58.33%	−0.743
3	29	−1.43%	−1.416	−1.36%	−1.349	58.33%	−0.743
4	29	−0.84%	−1.33	−1.46%	−1.428	58.33%	−0.743
5	29	−2.52%	−1.178	−1.08%	−0.875	65.52%	−1.486
6	29	−2.09%	−0.941	−0.65%	−0.411	58.33%	−0.743
7	29	−2.44%	−0.66	−0.14%	0.071	55.17%	−0.371
8	29	−2.29%	−0.335	0.48%	0.206	55.17%	−0.371
9	29	−1.73%	−0.66	−0.10%	−0.042	51.72%	0
10	29	−1.66%	−0.638	−0.75%	−0.327	51.72%	0
(0,1)	29	−0.24%	−0.962	−1.11%	−1.118 *	51.72%	0
(0,5)	29	−3.78%	−1.503	−6.61%	−1.435	58.33%	−0.743
(6,10)	29	−8.37%	−0.681	−0.88%	−0.086	58.33%	−0.743
(0,10)	29	−21.08%	−0.854	−7.49%	−0.524	58.33%	−0.743
Panel B. Non-heavy pollution industry							

** *p* ≤ 0.05; * *p* ≤ 0.10. ^a^ *Z*-statistics for medians were obtained using Wilcoxon signed-rank tests. ^b^ *Z*-statistics for % negatives were obtained using binomial sign tests. Note: Event Day 0 denoted the date of the announcement of environmental penalties.

**Table 6:** Descriptive statistics and correlations.

	M	S.D.	1	2	3	4	5	6	7	8	9	10
Age	19.489	4.528										
Firm sizes	9.818	15.992	0.124									
Sales	5.904	11.472	0.051	0.552 **								
Net profit	5.496	0.523	0.037	−0.058	−0.046							
Punished numbers	1.421	1.036	0.215 *	0.141	0.078	−0.043						
Ownership type	0.38	0.487	0.276 **	0.295 **	0.328 **	−0.082	0.231 *					
Environmental penalty	0.046	0.267	0.17	0.15	0.249 *	−0.018	0.733 **	0.206				
Investor attention	0.255	0.471	−0.067	−0.002	0.02	−0.016	−0.250 *	−0.044	−0.279 **			
Air pollution	0.067	0.368	−0.032	−0.064	−0.138	0.087	−0.109	−0.043	−0.178	0.036		
Industry saliency	0.67	0.473	0.033	0.175	0.028	−0.152	−0.066	−0.056	−0.220 *	0.157	−0.024	
Abnormal return	−0.006	0.034	0.043	−0.091	0.171	−0.059	0.023	0.245 *	0.01	0.206	−0.269 *	0.102

Note: *p* ≤ 0.05; *p* ≤ 0.10.

**Table 7:** Results of Regression Analyses.

Variables	Abnormal Returns	Abnormal Returns	Investor Attention	Investor Attention	Investor Attention	Investor Attention	Investor Attention
	Model1	Model2	Model3	Model4	Model5	Model6	Model7
Age	0.006	0.008	−0.012	−0.012	−0.013	−0.02	−0.018
Total assets	−0.318 *	−0.318 *	−0.002	−0.002	−0.001	−0.025	−0.006
Sales	0.287 *	0.269 *	0.088	0.087	0.095	0.085	0.073
Net profit	−0.043	−0.039	−0.02	−0.019	−0.018	−0.003	−0.001
Punished numbers	0.073	0.089	−0.076	−0.076	−0.049	−0.097	−0.121
Ownership type	0.248 **	0.248 **	−0.003	−0.003	−0.011	0.011	0.032
Environmental penalty	−0.12	−0.068	−0.279 **	−0.243 **	−0.282 **	−0.200 **	−0.251 **
Investor attention		0.214 *					
Air pollution				−0.002	−0.014		
Industry saliency						0.109	0.015
Environmental penalty * Air pollution					0.274 *		
Environmental penalty * Industry saliency							−0.184 **
$R^{2}$	0.142	0.181	0.09	0.079	0.075	0.1	0.095
Adjusted $R^{2}$	0.067	0.101	0.01	0.046	0.064	0.009	0.062
*F*	1.894 *	2.226 **	7.280 **	3.608 **	6.976 **	1.098 *	2.929 **

Standard errors are in parentheses. ** *p* ≤ 0.05; * *p* ≤ 0.10.

**Table 8:** The results on penalty amount and investor attention.

Panel A. Heavy pollution industry							
	N	**Median**	Z ^**a**^	**Mean**	t	**% Negative**	Z ^**b**^
Environmental penalty	59	0.16%	−4.731 ***	0.55%	1.836 *	8.48%	−6.093 ***
Investor attention (0)	59	1.50%	−3.550 ***	15.62%	3.670 ***	20.34%	−3.846 ***
Investor attention (1)	59	1.82%	−3.550 ***	30.70%	1.557	20.34%	−3.846 ***
Panel B. Non−heavy pollution industry							
	N	**Median**	Z ^**a**^	**Mean**	t	**% Negative**	Z ^**b**^
Environmental penalty	29	1.18%	−2.960 **	12.95%	1.526	17.24%	−3.213 ***
Investor attention (0)	29	6.68%	−1.784 *	9.19%	1.19	31.04%	−1.857 *
Investor attention (1)	29	11.61%	−2.206 **	15.05%	1.714 **	27.59%	−2.228 **

All tests are two-tailed: *** *p* ≤ 0.01; ** *p* ≤ 0.05; * *p* ≤ 0.10. ^a^ Z-statistics for medians were obtained using Wilcoxon signed-rank tests. ^b^ Z-statistics for % negatives were obtained using binomial sign tests.

## Data Availability

The data presented in this study are available on request from the corresponding author. The data are not publicly available due to privacy.
